# Supplementary material for: Vaccine Acceptance and Hesitancy among Hospitalized COVID-19 Patients in Punjab, Pakistan
Source: Vaccines (Basel). 2022 Sep 30;10(10):1640. doi: 10.3390/vaccines10101640 (PMC9611380; doi:10.3390/vaccines10101640)
Supplement: Supplementary file 1 [file vaccines-10-01640-s001.zip › vaccines-1880193-supplementary.pdf]

Table S1: **KMO and Bartlett's Test**

**Kaiser-Meyer-Olkin Measure of Sampling Adequacy.**		0.941
**Bartlett's Test of Sphericity**	Approx. Chi-Square	6069.913
	df	55
	Sig.	0.000

Table S2: Factors loading

	Factor	
**Questionnaire items**	**Hesitancy in receiving COVID vaccine**	**Trust on COVID vaccine**
I want to take the COVID vaccine because the vaccine is an effective way of protecting people from COVID infection	**0.885**	0.353
I want to take the COVID vaccine because the vaccine is important for my health and the health of my family and friends	**0.904**	0.348
I want to take the COVID vaccine because all COVID vaccines offered by the government are beneficial	**0.793**	0.432
I want to take the COVID vaccine because the vaccine lowers the risk of COVID infection	**0.906**	0.317
I want to take the COVID vaccine because vaccines prevent COVID infection from becoming worse, and the need for hospitalization	**0.896**	0.336
I want to take the COVID vaccine because the vaccine is a more effective preventive measure than using natural or other remedies	**0.788**	0.404
I want to take the COVID vaccine because COVID vaccines are as safe as other vaccines (e.g., flu vaccine)	**0.903**	0.348
I want to take the COVID vaccine because all COVID vaccines offered by the government are safe	**0.644**	0.494
I trust COVID vaccines because the benefits of the COVID vaccine outweigh their risk	0.461	**0.753**
I trust COVID vaccines because there are not many adverse effects reported for the COVID vaccine	0.295	**0.902**
I trust COVID vaccines because sufficient information is available about the long-term safety and efficacy of COVID vaccines	0.328	**0.881**

Extraction Method: Principal Component Analysis.
Rotation Method: Varimax with Kaiser Normalization.
a. Rotation converged in 3 iterations.

Table S3: Agreement or disagreement to study questionnaire items by vaccination status

Items	Non-vaccinated COVID patients		Vaccinated COVID patients		p-value
	Agreement n (%)	Disagreement n (%)	Agreement n (%)	Disagreement n (%)	
**1**. I want to take the COVID vaccine because the vaccine is an effective way of protecting people from COVID infection (n=380)	188 (49.5)	131 (34.5)	26 (6.8)	35 (9.2)	0.019
**2**. I want to take the COVID vaccine because the vaccine is important for my health and the health of my family and friends (n=382)	194 (50.8)	126 (33.0)	28 (7.3)	34 (8.9)	0.024
**3.** I want to take the COVID vaccine because all COVID vaccines offered by the government are beneficial (n=377)	165 (43.8)	150 (37.8)	25 (6.6)	37 (9.8)	0.083
**4.** I want to take the COVID vaccine because the vaccine lowers the risk of COVID infection (n=383)	195 (50.9)	126 (32.9)	27 (7.1)	35 (9.1)	0.012
**5.** I want to take the COVID vaccine because vaccines prevent COVID infection from becoming worse and the need for hospitalization (n=382)	195 (51.0)	124 (32.5)	28 (7.3)	35 (9.2)	0.014
**6.** I want to take the COVID vaccine because the vaccine is a more effective preventive measure than using natural or other remedies (n=381)	163 (42.8)	155 (40.7)	24 (6.3)	39 (10.2)	0.056
**7.** I want to take the COVID vaccine because COVID vaccines are as safe as other vaccines (e.g., flu vaccine) (n=383)	192 (50.1)	128 (33.4)	29 (7.6)	34 (8.9)	0.040
**8.** I want to take the COVID vaccine because all COVID vaccines offered by the government are safe (n=379)	148 (39.1)	168 (44.3)	24 (6.3)	39 (10.3)	0.203
**9.** I trust COVID vaccines because the benefits of the COVID vaccine outweigh their risk (n=378)	156 (41.3)	159 (42.1)	23 (6.1)	40 (10.6)	0.059
**10.** I trust COVID vaccines because there are not many adverse effects reported for the COVID vaccine (n=376)	141 (37.5)	174 (46.3)	31 (8.2)	30 (8.0)	0.385
**11.** I trust COVID vaccines because sufficient information is available about the long-term safety and efficacy of COVID vaccines (n=376)	134 (35.6)	175 (46.5)	32 (8.5)	29 (7.7)	0.192

Agreement = strongly agree or agree; disagreement=strongly disagree and disagree

Table S4: COVID-19 vaccines approved and available in Pakistan

Name of vaccine	Source	Date available in Pakistan for public use
Sinopharm	China	01-Feb-2021
Sinovac	China	23-May-2021
CanSino	China	31-Mar-2021
Oxford-AstraZeneca	WHO COVAX	08-May-2021
Pfizer	WHO COVAX	27-Aug-2021
Moderna	WHO COVAX	04-Jul-2021
Pakvac	Locally manufactured	02-Jun-2021
Sputnik V	Russia	03-Apr-2021
